# Debate: Are we overpathologising young people's mental health? Research shows otherwise – mental health conditions are not being recognised or diagnosed in healthcare settings

**DOI:** 10.1111/camh.70019

**Published:** 2025-07-31

**Authors:** Kapil Sayal, Rachel Hiller

**Affiliations:** ^1^ Institute of Mental Health, School of Medicine University of Nottingham Nottingham UK; ^2^ Division of Psychology & Language Sciences University College London London UK

## Abstract

Over recent years, there have been increasing societal, political and media concerns in relation to the ‘over‐diagnosis’ and ‘self‐diagnosis’ of common mental health conditions or emotional disorders, such as depression and anxiety. Using two large research projects as illustrative examples, we highlight that there is a mismatch between concern about ‘over‐pathologising’ young people's mental health and the recognition and diagnosis of emotional mental health conditions in health and care settings. Concerns around labelling risks us losing a shared understanding and language around mental health and mental health care, within services, between sectors, and for young people and families.

Diagnostic labelling of having a mental health condition or a disorder (diagnostic classification systems tend to use the term ‘disorder’ but many young people prefer the term ‘condition’) can be associated with both benefits and harms. In particular, there are risks from labelling if this is decoupled from the provision of evidence‐based support and interventions. A cautionary example comes from a 5‐year follow‐up of a school‐based 2 × 2 factorial trial that investigated outcomes following screening for attention‐deficit/hyperactivity disorder (ADHD) behaviours, with or without (a) provision of an educational intervention for teachers and/or (b) identification of high scorers (Sayal et al., 2010). The follow‐up found worse outcomes for the group who were identified to teachers as having high levels of ADHD behaviours but without the provision of the educational intervention (Sayal et al., 2010).

In this article, we primarily highlight two large‐scale research projects as illustrative examples of the mismatch between concern about ‘over‐pathologising’ young people's mental health and what the research evidence shows. One project focussed on children and young people referred to Child and Adolescent Mental Health Services (CAMHS) and the other on children with experience of the care system, referred for mental health support across CAMHS and social care settings.

## Shifts in diagnostic gatekeepers

The recently completed ‘STADIA’ trial investigated the effectiveness of a standardised diagnostic assessment tool in relation to 1225 children and young people with emotional difficulties who had been referred to CAMHS in England, and followed them up over 18 months (Sayal et al., 2025). What was particularly striking was that although 67% scored very high on a validated standardised diagnostic assessment (SDA) tool for at least one diagnosable emotional condition – most commonly depression or an anxiety disorder – only 11% received a clinical diagnosis of an emotional disorder from the service. This likely reflects a considerable amount of underidentification of emotional difficulties and diagnoses where thresholds are met, with potential consequences for evidence‐based treatments and interventions being offered.

Similarly, in the context of children's social care – where we know there are high rates of diagnosable mental health conditions – a growing body of evidence, including the England‐wide ADaPT project on best‐evidenced mental health care, has highlighted the pervasive underdetection of mental health conditions in this population, with a reliance of more generic terms such as ‘attachment problems’ or ‘trauma’ symptoms and an underdetection of common and trauma‐specific mental health conditions (McGuire, Halligan, Meiser‐Stedman, Durbin, & Hiller, 2022; Woolgar & Baldock, 2015). The potential consequences for access to services are stark – with high referral rejection rates (Coughlan et al., 2024), conceptualising mental health needs as ‘social care problems’, and a general lack of provision for evidence‐based mental health interventions for this group (McGuire et al., 2025).

A major concern is that neglecting carefully considered and accurate *diagnoses and formulations*, built on evidence‐based conceptualisations of mental health, will inevitably impact access to best‐evidenced mental health treatments and interventions. An additional knock‐on effect of this is that young people, in the absence of clear information and professional diagnosis, may seek to self‐diagnose (Underhill & Foulkes, 2025). We do not know the full consequences of the culture of self‐diagnoses for young people – but would we prefer a teenager to self‐diagnose as having bipolar disorder, for example or to have a trained mental health professional talk them through their depression or anxiety disorder in a way that minimises stigma and increases understanding?

## Conceptualisation of risk factors and difficulties

Clinicians/practitioners appear to commonly avoid making mental health diagnoses where children have significant or complicated life experiences – such as a history of trauma and adversity or where there may be ongoing instability or complicated circumstances. Yet, it is not incompatible to have difficult life events or trauma, on the one hand, and to have a diagnosis of a mental health condition such as depression or anxiety that may benefit from treatment. If we consider an example from physical health care, a child may have breathing difficulties (such as breathlessness or wheezing) with a contributory or exacerbating factor being exposure to parental smoking in the home. Of course, interventions to mitigate this should ideally include a shared understanding of the difficulties and attempts to stop exposure to second‐hand smoking. However, if the respiratory symptoms and their associated impact on the child's day‐to‐day life and functioning are sufficiently severe, withholding a diagnosis of asthma or the provision of an evidence‐based intervention for this would not be appropriate. In a similar way, we should not deny access to understanding and treatment/intervention for an identifiable and diagnosable mental health condition, because of life's complexities. The most important considerations are the child's needs in the here and now, how their mental health difficulties impact their functioning and well‐being, and what support can be provided.

## Diagnosis versus formulation

CAMHS is a specialist secondary healthcare service, with unique expertise in assessment, formulation, diagnosis, intervention and care delivery. Professionals within these services are under considerable pressure, with ever increasing need. However, even in the face of increasing pressures, it remains important that services strive to conduct high‐quality assessments and formulations (including considering and/or ruling out potentially eligible diagnoses), to ensure we are matching need to support and we are maximising the potential for positive outcomes for young people. It is crucial that commissioners recognise this too. Yet, multiple qualitative studies have shown that clinicians often prioritise formulation‐only approaches that do not incorporate potential and relevant diagnoses (McGuire et al., 2025; Thomson et al., 2025). Concerns around stigma, blame, ‘labelling’ and ‘overmedicalisation’ were often discussed.

In contrast, young people and parents/caregivers have expressed preference for diagnosis, as a first step to facilitate access to the right help (Thomson et al., 2025). This suggests a mismatch between clinician/practitioner views and the views of children and caregivers, which risks impacting clinical outcomes and engagement. Stigma may of course be a consideration and form part of a discussion with the child and caregiver. Soberingly, the ‘STADIA’ trial findings indicate that 12‐month clinical outcomes were poor for many children and young people across a range of domains, suggesting that their (significant) mental health needs were not fully identified. This also suggests that current practice approaches are often not meeting needs.

## Towards a shared understanding…

There is currently a huge amount of political and societal concern around ‘overdiagnosis’ of mental health disorders. However, in contrast to this concern, the available evidence suggests we are underdiagnosing mental health conditions, with potential implications for practice and outcomes. Even in specialist clinical services, we are frequently avoiding framing significant and impairing mental health difficulties in terms of diagnostic terminology (and the necessary and important conversation around this). This risks us losing a shared understanding and language around mental health and mental health care, within services, between sectors, and for young people and families.

For clarity, this article is not advocating that *all* young people entering mental health services need a diagnosis *all* the time. However, all children referred to mental health services – by definition – are struggling with their mental health. They know they are struggling. Their families know they are struggling. Young people's needs should be addressed in an evidence‐based way, considering symptoms and functioning, and both formulation and diagnosis – to facilitate access to psycho‐education (in a way that addresses concerns around stigma) and treatment planning. Young people are more mental health literate than ever before. To be truly collaborative, let us ensure they have a say in whether or not a diagnosis would be useful for their circumstances.

## Conflict of interest

Prof. Sayal is an editor of ‘Child and Adolescent Mental Health’.

## Ethics statement

Not applicable to this Debate article.

## Data Availability

Data sharing not applicable to this article as no data sets were generated or analysed.
